# Sugammadex to Facilitate Neurologic Assessment in Severely Brain-Injured Patients: A Retrospective Analysis and Practical Guidance

**DOI:** 10.7759/cureus.30466

**Published:** 2022-10-19

**Authors:** Sara J Hyland, Punit A Pandya, Cameron J Mei, David C Yehsakul

**Affiliations:** 1 Department of Pharmacy, OhioHealth Grant Medical Center, Columbus, USA; 2 Department of Neurosurgery, OhioHealth Grant Medical Center, Columbus, USA; 3 Department of Anesthesia, OhioHealth Grant Medical Center, Columbus, USA

**Keywords:** emergency neurosurgery, rocuronium, non-depolarizing neuromuscular blocking agents, rapid sequence intubation, neuroprognostication, intracranial hemorrhage, neuromuscular blockade, neurologic assessment, traumatic brain injury, sugammadex

## Abstract

Background

Widely used in anesthetic management, sugammadex is increasingly employed in the reversal of neuromuscular blocking agents (NMBAs) in the emergency department and critical care arena, where little evaluative data currently exists. This study explored the utility and safety of using sugammadex to facilitate neurologic assessments in critically ill, NMBA-exposed patients.

Methods

We pursued a retrospective case series and single-arm cohort analysis of all brain-injured patients receiving sugammadex to facilitate neurologic evaluation during one year at a high-volume Level 1 trauma center. The primary outcome was the qualitative impact of sugammadex administration on neurosurgeon decision-making. Secondary outcomes included the change to Glasgow Coma Scale (GCS) and hemodynamic parameters compared before and after sugammadex administration. Sugammadex dosing was also assessed across various weight scalars to explore dose-response trends and generate preliminary guidance for use in this setting.

Results

Our study criteria yielded 12 sugammadex administrations across 11 patients, the majority of whom had sustained a traumatic brain injury. All sugammadex administrations were adjudicated as beneficial to neurosurgeon decision-making and 50% were associated with a change to prognosis and plan. Sugammadex was associated with an increase in the GCS of 1-8 points among the 67% of patients who responded. Mean arterial pressure decreased significantly after sugammadex administration (median 94 vs. 104 mmHg, *p*=0.0215, median change of -8 mmHg [95%CI -25-3 mmHg]). No apparent dose-response trends were observed for changes to GCS or hemodynamic parameters.

Conclusions

The use of sugammadex to facilitate neurologic assessment after NMBA exposure in brain-injured patients was frequently associated with clinically meaningful changes to the neurologic exam and treatment plan. The risks of hemodynamic compromise and care complexity should be collaboratively weighed before pursuing this modality. An empiric sugammadex dose of 200 mg appears reasonable for this purpose, but further evaluation of NMBA reversal in the neurocritically ill outside of procedural settings is warranted.

## Introduction

Sugammadex is a novel chelating medication for non-depolarizing aminosteroidal neuromuscular blocking agents (NMBAs) and is indicated for the reversal of paralysis caused by rocuronium or vecuronium [1-3]. While sugammadex has been used routinely by anesthetists across the globe for over a decade, it has more recently been considered in the emergency department (ED) and intensive care unit (ICU) settings to facilitate timely neurologic assessments in patients who have received NMBAs, such as after rapid sequence intubation (RSI) for severe traumatic brain injury (TBI). This approach has merit in facilitating a more efficient and accurate neurologic assessment as compared to delaying the exam for NMBA clearance or proceeding with care plans despite the possibility of residual neuromuscular blockade, which persists longer than clinicians discern [2,4]. Additionally, detecting critical neurologic worsening in hospitalized TBI patients is imperative to initiating emergent management, and delays in the intervention (such as those that could be incurred by waiting for the spontaneous return of neuromuscular function after NMBA administration) are associated with increased mortality [5,6]. Hastening neurologic diagnosis, prognosis, and treatment of the critically brain-injured could confer significant benefits to patients, providers and hospitals.

To date, very limited studies have described patient outcomes and clinical considerations associated with sugammadex use outside of anesthesia settings [7-12]. While known to be well-tolerated across diverse surgical populations [13,14], sugammadex administration carries an infrequent risk of severe bradycardia, hypotension, and even asystole [15-18]. These risks may be more prevalent and deleterious in the neurocritically ill than in the elective surgical populations in which sugammadex has been studied and need to be better understood before this practice can be recommended routinely. The purpose of this study is to assess the use of sugammadex to facilitate neurologic assessment in brain-injured patients previously exposed to NMBAs at a high-volume level 1 trauma center and to provide practical guidance to neurocritical care clinicians pursuing this modality. This work was previously presented as a meeting abstract at the 2022 American College of Clinical Pharmacy Virtual Poster Symposium on May 25, 2022.

## Materials and methods

Institutional review board approval was obtained for this study (protocol number approval number 1781657) and we used the STROBE checklist when writing our report [19]. Our specific aims were 1) to describe patient circumstances and clinical outcomes associated with non-operative use of sugammadex to facilitate neurologic assessment in the ED and ICU settings at our center, and 2) to integrate our experience with a comprehensive review of the literature to generate practical guidance to be considered by clinicians and explored in future studies.

We pursued a retrospective, single-center, case series and single-arm cohort analysis of all patients who received sugammadex to facilitate neurologic assessment by the neurosurgical service at our high-volume tertiary care and Level 1 trauma center over the course of one year. All adult patients administered sugammadex outside of perioperative locations from July 1, 2019 to June 30, 2020 were screened for inclusion using electronic medical record data reporting. Both TBI and spontaneous intracranial hemorrhage (ICH) patients were eligible for inclusion. We excluded patients who were pregnant, those who did not receive an aminosteroidal NMBA prior to sugammadex administration, and those who were administered sugammadex for indications other than for the purpose of neurologic assessment (e.g., routine postoperative reversal or “can’t intubate, can’t ventilate” airway emergencies).

Sugammadex administration was demographically classified as facilitating initial neurologic assessment in the ED or facilitating postoperative neurologic assessment in the ICU. Patient case details, concomitant medications, neurologic assessments including the Glasgow Coma Scale (GCS), and hemodynamic variables surrounding the administration of sugammadex were collected via electronic extraction and manual chart review. The primary clinical outcome assessed was the impact of sugammadex administration on neurosurgeon decision-making. This designation was adjudicated retrospectively by a single resident neurosurgeon involved in the care of the patients and was described qualitatively as follows: change to the neurologic exam, change to the neurologic prognosis, change in the certainty of prior neurologic prognosis, change in decision to pursue further neuroimaging and/or other escalations of care, change to the neurosurgical treatment plan, or no discernible impact on neurosurgical decision-making.

Secondary clinical outcomes included change in blood pressure (BP), mean arterial pressure (MAP), and heart rate (HR) associated with sugammadex administration, the incidence of new or worsened hypotension (defined as a mean arterial pressure less than 65 mmHg) or bradycardia (defined as a heart rate less than 60 beats per minute), and incidence of peri-administration cardiopulmonary arrest. Hemodynamic parameters used in these assessments were garnered from the medical record using the last documented vital signs prior to sugammadex administration and the first documented vital signs after sugammadex administration, as long as these occurred within two hours of administration. Hospital course details including neuromonitoring device placement, neurological procedures, intracranial pressure (ICP) trends, length of stay, in-hospital mortality, and discharge disposition were also collected demographically. Additionally, details of sugammadex administration were collected and dose response was assessed in terms of various patient weight scalars, i.e., total body weight (TBW) and ideal body weight (IBW) via the Devine method [20].

A sample size determination was deemed beyond the scope of this project given the limited anticipated patients and single-arm design. To this end, we pursued a convenience sample of all patients meeting study criteria in the designated timeframe. Based on pharmacy medication order data regarding the rate of sugammadex administrations outside of perioperative locations, our case series sample was expected to not exceed 20 patients. Demographic variables, medication data, and primary clinical outcomes were summarized descriptively using frequencies and percentages for nominal variables and medians with interquartile or total ranges for ordinal and continuous variables. For the secondary clinical outcomes, two-sided, nonparametric confidence intervals were determined for the median changes in patient GCS, BP, and HR. These variables were also compared from post- to pre-sugammadex administration via the Wilcoxon Signed Rank Test (tests the hypothesis that the distribution of the difference has a median of zero) with alpha set at 0.05. Patients for whom complete GCS and vital sign data were not documented within the pre-specified timeframe relative to sugammadex administration were excluded from these secondary analyses. Pre-specified subgroup analyses included comparing secondary clinical outcomes among sugammadex “responders,” i.e., those who experienced a change in GCS after administration, suggesting residual neuromuscular blockade was masking a (wholly or partially) neurologically intact patient, and among “non-responders,” i.e., those for whom GCS was unchanged after sugammadex administration. Lastly, sugammadex dose was also assessed in terms of weight-based and non-weight-based dosing scalars for correlation to changes in GCS, MAP, and HR using scatter plots and simple linear regression to generate coefficients of determination (R2 values via the method of least squares).

## Results

Demographic outcomes

**Figure 1:**
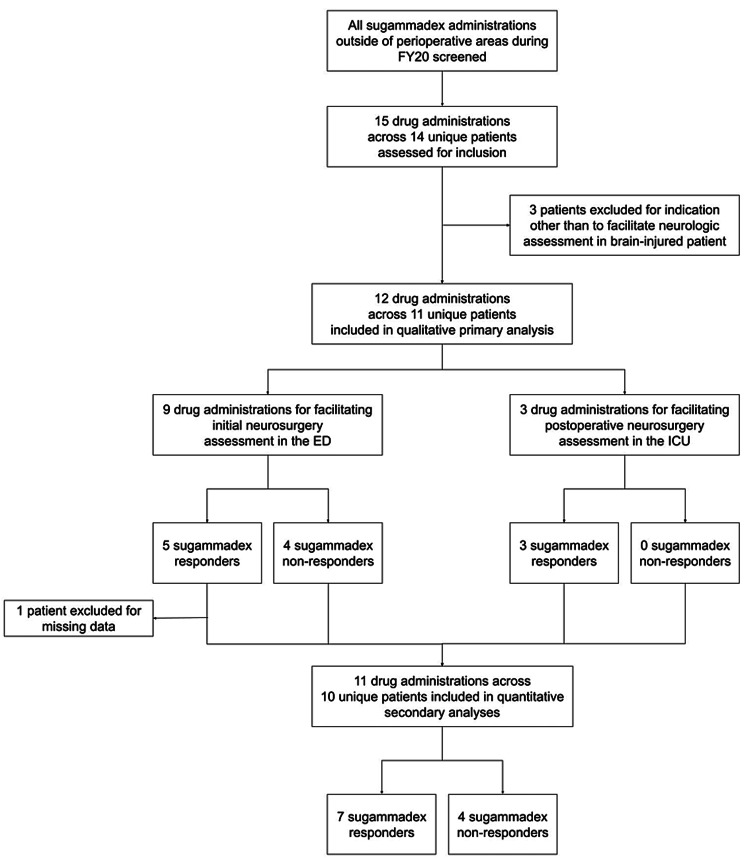
Population determination ED=emergency department, FY20=fiscal year 2020 (July 1, 2019 to June 30, 2020), ICU=intensive care unit

Our initial screening criteria indicated 15 total sugammadex administrations across 14 unique patients during the pre-specified timeframe (Figure 1). Three of these were excluded for sugammadex indications other than reversing residual neuromuscular blockade to facilitate neurological assessment in a brain-injured patient. This yielded 12 sugammadex administrations across 11 patients included in the final qualitative primary analysis, nine (75%) administrations having been pursued initial neurologic assessments in the ED and three (25%) for postoperative neurologic assessments in the ICU.

Summative case information for each included patient and sugammadex administration is displayed in Table 1. The majority of patients (8/11) had sustained traumatic brain injuries while fewer (3/11) had experienced spontaneous ICH. The population was predominantly male (77%) with a median age of 69 years (range 23 to 79). Seven (58%) sugammadex administrations followed rocuronium as the antecedent NMBA, four (33%) administrations followed vecuronium, and one administration followed both rocuronium and vecuronium exposures. No other non-depolarizing neuromuscular blockers were administered to these patients prior to pursuing reversal with sugammadex and neostigmine was not administered to any of these patients. 

**Table 1:** Case series summary Legend: BtO_2_=brain tissue oxygen, CT=computed tomography, ED=emergency department, EEG=electroencephalogram, EVD=external ventricular drain, GCS=Glasgow Coma Scale, GSW=gunshot wound, intraop=intraoperatively, ICH=intracerebral hemorrhage, ICP=intracranial pressure, ICU=intensive care unit, IPH=intraparenchymal hemorrhage, IPR=inpatient rehabilitation, IVH=intraventricular hemorrhage, LTAC=long-term acute care, LUE=left upper extremity, MCA=middle cerebral artery, MCC=motorcycle collision, MRI=magnetic resonance imaging, MVC=motor vehicle collision, neuro=neurologic, NMPT=nuclear medicine perfusion test, OR=operating room, Roc=rocuronium, RSI=rapid sequence intubation, SAH=subarachnoid hemorrhage, SDH=subdural hematoma, SNF=skilled nursing facility, TBI=traumatic brain injury, Vec=vecuronium

Patient Case Number	Patient Presentation	Details of NMBA Administration	Details of Sugammadex Administration	Neurologic Exam Change After Sugammadex Administration	Clinical Decision -Making After Sugammadex Administration	Patient Course
1a	72yof with nontraumatic IVH with obstructive hydrocephalus	Roc 100 mg for RSI in ED	200 mg (1.8 mg/kg) for initial assessment in ED	Exam improved significantly (GCS 3T to 11T)	Changed neurosurgery plan - EVD was placed and ultimately surgery when intervention may not have been pursued	EVD placed in ED then patient to OR for burr hole craniotomy with IVH evacuation on hospital day 2; steady postop progress and discharged to LTAC on hospital day 24, discharged to SNF thereafter
1b		Roc 50 mg then Vec 2 mg intraop for craniotomy	200 mg (1.8 mg/kg) for postop assessment in ICU	Exam improved significantly (GCS 3T to 11T)	Avoided unnecessary testing and/or escalations of care (e.g. CT, MRI, EEG)	
2	71yom with nontraumatic IPH	Vec 10 mg intraop for craniotomy	150 mg (1.6 mg/kg) for postop assessment in ICU	GCS improved significantly (3T to 10T), started following commands	Avoided unnecessary testing and/or escalations of care (e.g. CT, MRI, EEG)	Discharged to IPR on hospital day 19
3	69yof with traumatic SDH with midline shift and SAH s/p fall	Roc unknown dose for RSI prior to arrival	170 mg (2.8 mg/kg) for initial assessment in ED	GCS unchanged at 3T but new brainstem reflexes emerged	Changed neurosurgery plan - operative intervention offered when intervention may not have been pursued	To OR for emergent craniectomy; ultimately expired hospital day 3 after comfort care measures instituted
4	51yom with severe TBI s/p unhelmeted MCC, large IPH	Vec 10 mg given prehospital	160 mg (1.8 mg/kg) for initial assessment in ED	GCS unchanged at 3T, exam remained poor despite 4/4 twitches on TOF, no cough or gag	Increased certainty of poor prognosis and affirmed plan for no neurosurgical intervention due to catastrophic injury	Family elected for comfort care measures and patient expired on hospital day 1
5	78yom acute on chronic SDH with midline shift s/p fall	Roc 50 mg dosed 3 times intraop (total dose 150mg)	290 mg (3.7 mg/kg) for postop assessment in ICU	Exam changed significantly (GCS 3T to 8T)	Avoided unnecessary testing and/or escalations of care (e.g. CT, MRI, EEG)	Discharged to IPR on hospital day 16
6	23yom with traumatic SDH and SAH with diffuse cerebral edema s/p MVC with ejection	Vec 8 mg prehospital	320 mg (3.7 mg/kg) for initial assessment in ED	GCS unchanged at 3T, exam remained poor	Increased certainty of poor prognosis and affirmed plan for no operative intervention	ICP monitor placed; brain death confirmed via NMPT, patient expired on hospital day 1
7	59yom with multifocal traumatic ICHs including IVH, IPH, SAHs s/p fall	Roc dosed multiple times prior to arrival (total 270 mg)	310 mg (3.9 mg/kg) for initial assessment in ED	GCS unchanged at 3T, exam remained poor	Increased certainty of poor prognosis and affirmed plan for no neurosurgical intervention	EVD placed; ultimately patient expired on hospital day 9 after comfort care measures instituted
8	53yof with extensive traumatic SAH and temporal IPH with IVH, hydrocephalus and midline shift s/p MVC	Roc 70 mg given in ED for RSI	280 mg (3.9 mg/kg) for initial assessment in ED	Exam improved significantly (GCS 3T to 8T)	Changed neurosurgery plan - EVD was placed and further imaging found MCA aneurysm, triggering transfer to neuroendovascular suite when intervention may not have been pursued	Emergently transferred to neuroendovascular intervention facility where aneurysm clipped and IPH evacuated; ultimately expired on hospital day 19 after comfort care measures instituted
9	71yom with large cerebellar IPH after being found down	Roc unknown dose given for RSI prior to arrival	200 mg (1.8 mg/kg) for initial assessment in ED	Exam improved significantly (GCS 3T to 7T)	Changed neurosurgery plan - operative intervention offered when intervention may not have been pursued	EVD placed and patient to OR for suboccipital craniectomy hospital day 1; developed multi-organ failure postop, ultimately expired on hospital day 7 after comfort care measures instituted
10	79yom with severe TBI s/p GSW to head, multifocal ICHs with skull fractures	Vec 10 mg given for RSI prior to arrival	500 mg (4.8 mg/kg) for initial assessment in ED	Exam slightly improved (GCS 3T to 4T) with return of brainstem reflexes, new LUE postering	Changed neurosurgery plan - operative intervention offered when intervention may not have been pursued	Family elected for comfort care measures and patient expired on hospital day 2
11	50yom with multifocal traumatic ICHs including IPH with skull fractures s/p unhelmeted MCC	Roc 120 mg given for RSI in ED	480 mg (4.0 mg/kg) for initial assessment in ED	Exam improved significantly (GCS 3T to 8T)	Changed neurosurgery plan - ICP and BtO_2_ monitors placed and operative intervention ultimately pursued when intervention may not have been pursued	ICP and BtO_2_ monitoring placed, patient to OR for decompressive craniectomy on hospital day 3 for persistently elevated ICPs and loss of cough reflex; ultimately patient expired on hospital day 8 after comfort care measures instituted

Clinical outcomes

For the primary outcome determination of the initial neurologic assessments occurring in the ED, six (67%) sugammadex administrations were associated with a change to the neurosurgeon prognosis and plan, and three (33%) were associated with increased certainty in the neurosurgeon prognosis, affirming the previous plan. Of the postoperative neurologic assessments occurring in the ICU, three (100%) were associated with avoidance of unnecessary testing and escalations of care. Hence, all sugammadex administrations in the study population were deemed beneficial to neurosurgeon decision-making and 50% of all administrations were associated with a change to neurologic prognosis and neurosurgery plan. Eight of the 11 total patients (73%) in this analysis ultimately expired during hospitalization, and no deaths appeared related to sugammadex administration: death occurred one to 18 days after sugammadex use, and all deaths were documented as being related to catastrophic brain injury with or without subsequent multiorgan failure.

**Figure 2:**
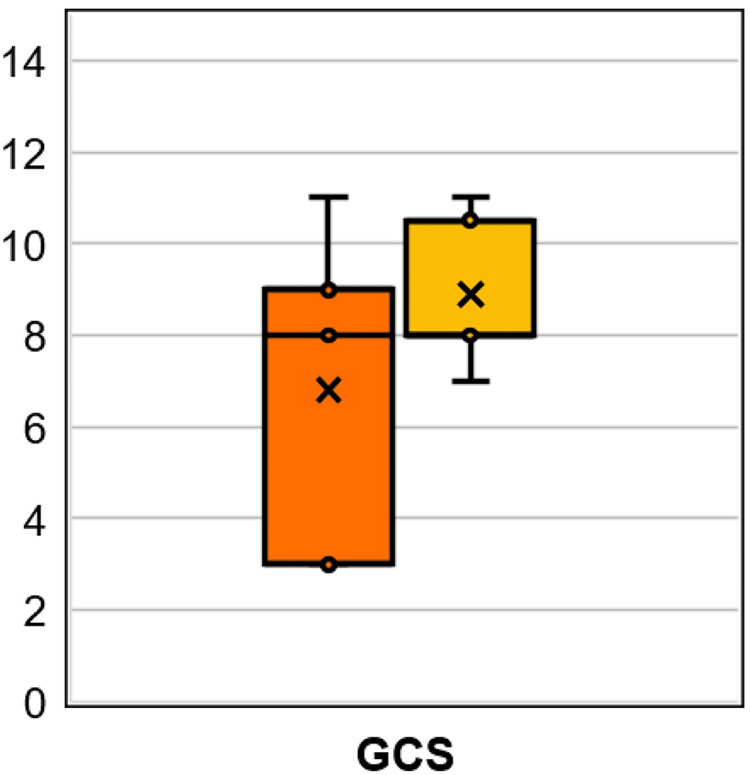
GCS after sugammadex administration All patients had a Glasgow Coma Scale (GCS) score of 3T prior to sugammadex administration. The distribution of GCS for the total population post-sugammadex is represented in the orange box-and-whiskers plot (n=11, p=0.0156 compared pre- vs. post-sugammadex), and for the sugammadex responders' subgroup in the yellow plot (n=7, p​​​​​​​=0.0156 compared pre- vs. post-sugammadex). Interpretation: Middle line inside box denotes median, x denotes mean, outer lines of box denote interquartile range, ends of whiskers denote total range (Note: values outside 1.5 times the interquartile range are considered outliers and instead represented by dots). P-values noted are from Wilcoxon Rank Sum Test (tests null hypothesis that the distribution of the differences has a median=0).

All patients exhibited a GCS of 3T prior to sugammadex administration, and GCS increased after sugammadex in 67% of drug administrations, yielding eight sugammadex “responders.” Of these, one patient chart was missing vital signs documentation within two hours post-sugammadex administration and so was excluded from the secondary analyses, resulting in a final n=11 (seven responders and four non-responders) for the secondary quantitative analysis. A significant increase in median GCS was observed after sugammadex administration in the total included population (8T vs. 3T, p=0.0156, median increase of five points [95% CI 0-8 points], n=11), inherently owing to the sugammadex responders' subgroup (8T vs. 3T, p=0.0156, median increase of five points [95% CI 4-8 points], n=7) (Figure 2).

Overall, MAP was lower after sugammadex administration in nine of 11 administrations (82%) and HR was lower after sugammadex in six of 11 administrations (55%). New or worsening bradycardia or hypotension only occurred in three (27%) administrations, however, and no major cardiopulmonary events were noted within two hours of sugammadex administration in this cohort. Of the three patients who experienced new or worsening hypotension or bradycardia after sugammadex administration, two expired (67% mortality), as compared to six of eight patients (75% mortality) who did not experience one of these events. Similarly, mortality was 5/9 (56%) in patients whose MAP decreased after sugammadex administration vs. 2/2 (100%) in patients whose MAP did not decrease.

**Figure 3:**
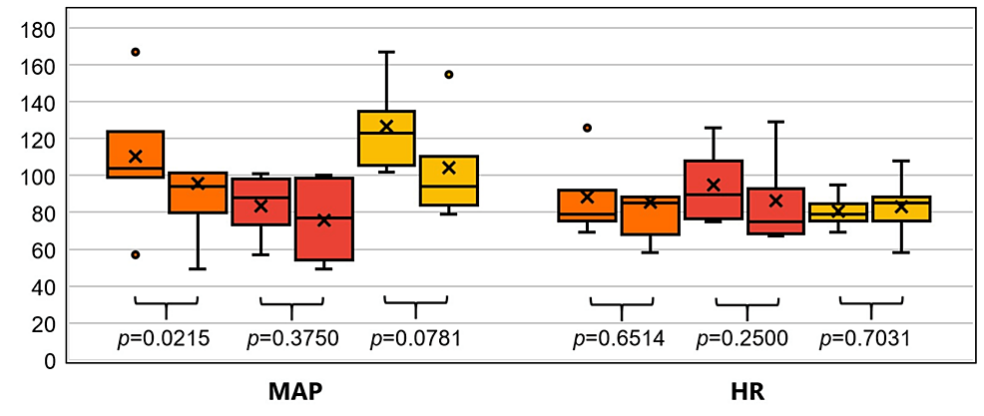
MAP and HR compared before and after sugammadex administration HR=heart rate in beats per minute, MAP=mean arterial pressure in millimeters of mercury. Orange box-and-whisker plots include total population (n=11), red plots represent the sugammadex non-responder subgroup (n=4), and yellow plots represent the sugammadex responder subgroup (n=7). Within each pair of plots, the plot on the left represents pre-sugammadex administration and the plot on the right represents post-sugammadex administration. Interpretation: Middle line inside box denotes median, x denotes mean, outer lines of box denote interquartile range, ends of whiskers denote total range (Note: values outside 1.5 times the interquartile range are considered outliers and instead represented by dots). P-values noted are from Wilcoxon Rank Sum Test (tests null hypothesis that the distribution of the differences has a median=0).

In the quantitative analysis, the median MAP in the total population decreased significantly after sugammadex as compared to prior to drug administration (94 vs. 104 mmHg, p=0.0215, median change of -8 mmHg [95%CI -25-3 mmHg]) (Figure 3). The sugammadex responder subgroup had higher median MAPs and larger MAP decreases in response to sugammadex (94 vs. 123 mmHg, median change -15 [95% CI -73-10], n=7) as compared to the sugammadex non-responder subgroup (77 vs. 88, median change -5.5 [95% CI -23-3], n=4), though the change in median MAP did not reach statistical significance within either subgroup (p=0.0781 and p=0.375, respectively). No significant changes in median HR were observed in response to sugammadex in the total population (85 vs. 79 bpm. p=0.6514, median change -6 bpm [95% CI -13-19], n=11) or in either subgroup individually (Figure 3).

Medication dosing outcomes

Sugammadex was administered at a median dose of 240 mg (range 150 to 500 mg) at a median time of 101 minutes after the last dose of antecedent non-depolarizing NMBA (range 42 to 231 minutes). Weight-based sugammadex doses ranged from 1.6 to 3.8 mg/kg TBW (median 3.25 mg/kg) and 1.9 to 7.3 mg/kg IBW (median 4.1 mg/kg). Changes in sugammadex dose, as assessed by any of the three studied dosing scalars, did not appear to predict the variation in change to GCS, MAP, or HR, as evidenced by very low R2 values ranging from 0-0.367 (Table 2).

**Table 2:** Regression analyses exploring dose-response trends for sugammadex effect on GCS, MAP, and HR Legend: GCS=Glasgow Coma Scale, HR=heart rate, IBW=ideal body weight, MAP=mean arterial pressure, mg=milligram, TBW=total body weight, R2=coefficient of determination (simple linear regression line via least squares method)

Sugammadex Dosing Scalar	Dose vs. Change in GCS	Dose vs. Change in MAP	Dose vs. Change in HR
Dose (mg)	R2=0.044	R2=0.093	R2=0.113
Weight-Based Dose (mg/kg TBW)	R2=0.184	R2=0.367	R2=0.014
Weight-Based Dose (mg/kg IBW)	R2=0	R2=0.126	R2=0.252

## Discussion

Sugammadex mediates an impressively fast and consistent erasure of neuromuscular blockade and may be associated with improved perioperative clinical outcomes, prompting exploration of its utility beyond the operative theater [8,21,22]. We sought to better quantify both the perceived benefits and unclear risks of employing sugammadex in patients presenting with TBI or non-traumatic ICH in the emergent department and critical care settings. All sugammadex administrations in this small retrospective study were deemed valuable to neurosurgeon decision-making and half of all administrations were associated with a change to neurologic prognosis and neurosurgery plan. Two-thirds of the population had an apparent response to sugammadex, suggesting residual neuromuscular blockade was interfering with the neurologic exam, and GCS increased from 3T to 4-11T in this subset. Mean arterial pressure was significantly reduced after sugammadex administration with a median reduction of 8 mmHg, though HR was not significantly altered. No apparent linear relationships could be inferred between sugammadex dose and changes to GCS, MAP, or HR when assessed by any of the three dosing scalars.

From the neurosurgeon's perspective, a neurological exam is the most basic and fundamental tool for patient evaluation. For example, when first encountering a patient with post-traumatic subdural hematoma with significant mass effect, it is essential to ascertain an accurate initial neurological exam. Patients with a poor initial neurological state, as indicated by a very low GCS, may not benefit from a neurosurgical intervention [23-26]. Prior to reaching such a conclusion and relaying a devastating prognosis, however, it is crucial to ensure the apparent neurological state of the patient is not compromised by any pharmacologic agents, such as NMBAs, which are commonly employed when securing the airway [23,27]. In such scenarios, rapid and reliable reversal of neuromuscular blockade with sugammadex may be vital to reveal the true GCS of the patient to inform care planning. If the GCS proves to be higher once residual neuromuscular blockade is removed, neurosurgeons may elect to proceed with surgical evacuation of the mass lesion. However, if the very poor mental state persists post-paralytic reversal, the recommendation for comfort measures may be pursued with greater certainty. Likewise, if a postoperative neurologic exam suggests unexpectedly depressed responses, it again becomes a question of whether residual neuromuscular blockade from surgical paralysis is interfering. If the GCS rapidly improves after sugammadex administration in such circumstances, then imaging and other escalations of care directed at determining an alternative cause may be avoided.

From the analysis of our data set, we conclude that prolonged residual paralysis after intubation interferes with neurologic exams with alarming frequency. This is evidenced by a positive GCS response being observed after sugammadex administration among patients who had their last antecedent NMBA dose administered 42-231 minutes prior, with the majority of these occurring after the oft-quoted one-hour duration of rocuronium/vecuronium. While this may seem unexpected, clinician-assessed duration of paralysis is likely to far exceed those reported in anesthesia texts since clinical trials defined NMDA duration as “median time to 25% recovery of first muscle twitch height” [28,29]. Real-world evaluation has previously validated that single intubating doses of intermediate-acting NMBAs commonly confer residual paralysis even more than two hours after administration [30]. Our analysis, therefore, aligns with prior evidence that residual neuromuscular blockade is common after intubation and is likely underestimated by clinicians [2,31]. To this end, we did find that an improved GCS score following the complete reversal of paralysis by sugammadex influenced patient care, and we support recommendations for its consideration in this setting [8,27].

These benefits were accompanied by evidence of adverse event risks. The significant reduction in MAP with sugammadex is an important finding that should not be overlooked by neurosurgical providers since maintenance of adequate blood pressure after brain trauma is vital for brain perfusion during periods of acute swelling. Current guidelines for the management of severe TBI advise maintaining systolic blood pressure ≥100-110 mmHg to decrease mortality and improve outcomes, with recommended target cerebral perfusion pressures of 60-69 mmHg to improve survival and favorable outcome [32]. Of the patients who were above their age-specific systolic blood pressure goal prior to sugammadex in our cohort, three (27%) fell below this threshold after sugammadex administration. The vast majority of our patients experienced some reduction in MAP, with this reduction ranging from 3-73 mmHg. Neurocritical care providers must therefore be acutely aware of potential hypotensive adverse effects with sugammadex and be ready to intervene if necessary.

We did not find a significant reduction in HR after sugammadex administration, which is an interesting finding given that sugammadex is known to cause transient bradycardia that may be more likely in vulnerable patients and at higher doses [13,15,18,33]. We hypothesize that any sugammadex-mediated bradycardia could have been counteracted by the concurrent patient stimulation incurred during the neurologic exams for which sugammadex was administered, and/or by the catecholamine surge that often accompanies acute TBI [34,35]. Bradycardia occurs on the order of 1% in general surgical populations receiving sugammadex [18] and is significantly less likely with sugammadex as compared to neostigmine [21]. Our results can be compared with another recent retrospective assessment by Hile et al., which assessed hemodynamic instability that required escalation of treatment within 30 minutes of sugammadex or neostigmine with glycopyrrolate administered to ED patients previously intubated with rocuronium [10]. This study did not identify any such events in the sugammadex group (n=10) and no significant difference between sugammadex and neostigmine groups with regard to the primary outcome (0% vs. 14.8%, p=0.557, total n=37) [10]. Conversely to our data, a 25% incidence of bradycardia, but no hypotension, was observed with sugammadex in the eight patients for which hemodynamic data were available. These authors also highlight that the safety of paralytic reversal in the ED has not been well described and further evaluation is needed [10].

In addition to the risk of negative hemodynamic effects, the use of sugammadex in this setting can complicate important components of neurocritical care. Subsequent indications for the neuromuscular blockade, such as surgical paralysis during emergent craniotomy, may become more challenging since larger doses of rocuronium or vecuronium will be required to overcome recent sugammadex exposure [13,36]. Alternatively, a benzylisoquinolinium non-depolarizing NMBA such as cisatracurium could be used for recurarization. However, because sugammadex does not bind this structural class, direct and rapid reversal may not be achievable, possibly delaying subsequent postoperative neurologic assessments. Sugammadex also artifactually interferes with coagulation assays including activated partial thromboplastin time (aPTT), prothrombin time (PT/INR), and R-time on thromboelastography (TEG) such that bleeding times may appear prolonged [13,37,38]. For these reasons, we recommend all involved care teams be made aware when a patient has received sugammadex.

Though the use of sugammadex in this setting is well within the labeled indication of reversing neuromuscular blockade from rocuronium or vecuronium, the tailoring of risk-benefit assessment, dosing, and monitoring to the neurocritically ill patient is warranted. Our present dataset is small, but we feel it is an accurate sample of severely brain-injured patients generalizable to those of other large trauma centers. In the absence of specific and evidenced-based recommendations for such patients, our institutional data and the aforementioned considerations were integrated with a thorough review of applicable literature by the multidisciplinary collaborators, whose expertise spans perioperative and emergency medicine clinical pharmacology, neurosurgery, and neuroanesthesia. Our guidance in Table 3 may support neurocritical care providers in making complex patient-specific assessments and lay the groundwork for future studies.

**Table 3:** Recommended clinical assessment and considerations for use of sugammadex to facilitate the neurologic assessment Legend: bpm=beats per minute, GCS=Glasgow Coma Scale, HR=heart rate, MAP=mean arterial pressure, mmHg=millimeters of mercury, NMBA=neuromuscular blocking agent

Assessment Question	Clinical Considerations and Recommendations	
Which NMBA(s) was(were) given previously?	Sugammadex cannot reverse neuromuscular blockade caused by succinylcholine or benzylisoquinolinum NMBAs such as atracurium or cisatracurium	Sugammadex should only be used to reverse neuromuscular blockade caused by rocuronium or vecuronium
How long ago was the NMBA administered, and at what dose/dosing strategy?	Sugammadex may not reverse neuromuscular blockade caused by continuous infusions of NMBA as predictably as for intermittent doses but may still be considered	Larger doses of sugammadex may be required to reverse neuromuscular blockade when administered shortly after NMBA administration
What is the value of removing residual neuromuscular blockade to this patient’s care?	Anticipate many patients experiencing a change in exam after sugammadex, and an increase in GCS of anywhere from 1-8 points among patients who do respond	Consider removing residual neuromuscular blockade to facilitate neurologic assessment if there is potential to cause an important change to neurosurgeon prognosis and plan for the patient
What are the risks of new or worsening hypotension and/or bradycardia in this patient?	Anticipate a reduction in MAP of approximately 8 mmHg after sugammadex administration, with a reduction by as much as 25 mmHg possible; a minority of patients may experience new or worsening hypotension (MAP<65 mmHg) or bradycardia (HR<60 bpm), though these events should be less frequent than with neostigmine plus glycopyrrolate	Sugammadex has also been rarely associated with bradycardic cardiovascular arrest and life-threatening anaphylaxis events – their frequency is very low but these can be devastating and difficult to treat
Will the patient require reintroduction of neuromuscular blockade after sugammadex administration?	Sugammadex renders subsequent rocuronium and vecuronium administration less effective for 4 hours (up to 24 hours in renal impairment), requiring higher doses or alternative NMBAs	If alternative agents such as atracurium or cisatracurium are used, the ensuing neuromuscular blockade will not be rapidly and completely reversible at all depths since neostigmine is their only available reversal agent at this time
How can we make this therapy most successful?	Account for sedatives and other narcotics that may require reversal and/or delay of neurologic exam before assessing for residual neuromuscular blockade and potential utility of its reversal; Use of train-of-four monitoring can add value to the assessment when available	Also consider the possibility of under-sedation being masked by residual neuromuscular blockade, and the potential consequences of unmasking this with sugammadex (e.g. agitation, pulling at lines/tubes); ensure supportive therapies are immediately available should hemodynamic or behavioral instability occur
Given the answers to the above, does the interprofessional team feel that sugammadex is indicated, and that the likely benefits of proceeding outweigh the potential risks in the patient?	An interprofessional and patient-specific assessment is advised that incorporates the above considerations before proceeding with sugammadex in neurocritically ill patients	Sugammadex administration should be documented and communicated to the entire care team, and preemptive consultation with Anesthesiology and/or Pharmacy is advised if the patient will require surgery or otherwise need subsequent paralysis after sugammadex
If we proceed, how should sugammadex be dosed in this setting?	A dose of 200 mg IVP could be considered for this indication in adult patients based on tailored treatment goals and risks; To facilitate this strategy, the commercially-available 200 mg vials could be made available in applicable critical care and emergency department areas and/or in emergency response pharmacist boxes depending on institutional practice model/procedures	Consideration could be given to repeating this dose after 5 minutes in select circumstances if an incomplete response is perceived (e.g. in patients who had received large quantities or continuous infusions of NMBAs, or when given within minutes of NMBA)
If we proceed, how should sugammadex be monitored in this setting?	Patients should be closely monitored in intensive care including continuous/frequent hemodynamic assessments and ventilator support as indicated; anaphylaxis should be suspected and treated aggressively should persistent unexplained hypotension, bronchospasm, urticaria and/or angioedema emerge	Recognize the potential for transient interference with coagulation assays such that bleeding times may appear prolonged

We believe a flat dose of 200 mg sugammadex by intravenous push is reasonable in this setting for several reasons. It is important to reiterate that the goal of sugammadex administration in our study differs from that in perioperative assessments; here paralytic reversal is undertaken to facilitate a more accurate neurosurgical assessment, whereas in most surgical publications sugammadex is used to facilitate extubation and avoid residual paralysis to protect post-extubation respiratory function. A low level of residual paralysis primarily affects the very small muscle groups such as those responsible for the pharyngeal function and intraocular movement, and we consider this to be largely inconsequential in the setting of assessing GCS in an intubated patient. Additionally, the manufacturer-recommended dosing of 2 mg/kg and 4 mg/kg actual body weight pertain to the reversal of moderate and deep levels of neuromuscular blockade, respectively [36]. At the time of neurosurgeon evaluation, which occurred 42 to 231 minutes after antecedent NMBA in our study, the neuromuscular block is likely to have fallen to shallower levels. This will vary and can only be confirmed with quantitative train-of-four monitoring. Sugammadex doses of 0.25-2 mg/kg have been recommended for reversing lighter depths across the minimal to moderate block range based on multiple dose-ranging studies [2,39,40]. A 200 mg dose achieves this range across diverse adult patient weights. Phase-II dose-finding evaluation also demonstrates comparable efficacy of 2 mg/kg and 4 mg/kg for reversing rocuronium given at 0.6-1.2 mg/kg doses, with full neuromuscular recovery being achieved by both doses approximately two minutes apart, on average [41]. These data suggest little additional therapeutic benefit would be gained above the 2 mg/kg dose for our population, for which post-anesthesia care unit throughput is not a concern. This aligns with our limited regression analyses, which found the proportion of variation in clinical effect that is predictable from sugammadex dose to be quite low. Lastly, though the manufacturer recommends the use of total body weight as the scalar for dosing to avoid the risk of treatment failure, this has been contested. Published validations of corrected or IBW scalars for sugammadex dosing in obese patients now exist and the use of IBW has been recommended in some guidelines [1,42-44]. The effectiveness of a flat 200 mg dose was anecdotally validated by patient #1 in our case series, a morbidly obese woman who experienced a nearly immediate animation of all extremities and a dramatic increase in GCS from 3T to 11T after a 200 mg (1.8 mg/kg) dose of sugammadex was given. This phenomenon occurred at each of two different points in her hospitalization. Similarly, Smack et al. report a case with nearly identical positive results in a 157 kg patient who received sugammadex 200 mg (1.27 mg/kg) only seven minutes after rocuronium administration for RSI [7]. Using a flat dosing approach in the presently studied setting, therefore, appears effective and sensible given the augmented treatment goals and limited risks posed by recurarization. This dosing scheme also facilitates efficient medication supply and preparation given the commercially available 200 mg/2 mL ready-to-administer vial size [36].

This study does suffer from a number of limitations. Our retrospective, single-arm design and small sample size precluded meaningful analyses as statistical power could not be assessed, limiting our methodology to hypothesis-generating in rigor. We had no capacity to incorporate an assessment of the depth of neuromuscular blockade before and after reversal as such monitoring is not routinely available in our ED, though this appears to be a commonplace practice opportunity. Additionally, while we assessed changes to hemodynamic parameters to the extent of available documentation, there was no standard interval between assessments in relation to sugammadex administration, and we did not assess for other indicators of severe adverse hemodynamic events such as atropine administration or the initiation of vasopressors, which could have resulted in under-reporting of potential risks with sugammadex. Still, a comprehensive review of medical documentation for our population suggested against the occurrence of significant medication-related morbidity or mortality, such as the known rare risks of cardiovascular collapse or anaphylaxis with sugammadex [13,45]. Furthermore, our assessment of sugammadex utility was adjudicated retrospectively by a single, unblinded neurosurgical resident, and would be strengthened by multiple blinded assessors in a prospective manner. Despite these limitations, our study is currently the largest of its kind, to our knowledge, and adds to the presently very limited literature supporting sugammadex to facilitate the neurologic assessment of neurocritically ill patients in the ED and ICU settings. Further study of sugammadex outside the perioperative setting in situations as presented here is warranted. Future studies should assess both the utility and safety of sugammadex in this population through prospective, standardized, blinded assessments.

## Conclusions

Residual neuromuscular blockade may frequently interfere with neurologic assessment after NMBA exposure in brain-injured patients in the ED and ICU, even more than an hour after NMBA administration. Trialing sugammadex in these situations may therefore benefit neurosurgical decision-making by removing residual neuromuscular blockade and facilitating timely, accurate neurologic exams. We recommend these benefits be weighed against the risks of lowering blood pressure and complicating subsequent care in collaborative decision-making when considering this modality. We feel an empiric sugammadex dose of 200 mg via intravenous push is reasonable for this purpose to balance benefits and risks. A more robust evaluation of sugammadex to facilitate neurologic assessment in neurocritically ill patients outside of procedural settings is warranted before this practice can be recommended routinely.
